# Using the RE-AIM framework to evaluate a community-based summer camp for children with obesity: a prospective feasibility study

**DOI:** 10.1186/s40608-015-0050-8

**Published:** 2015-05-14

**Authors:** Shauna M Burke, Sheree Shapiro, Robert J Petrella, Jennifer D Irwin, Michelle Jackman, Erin S Pearson, Harry Prapavessis, Joel Kevin Shoemaker

**Affiliations:** School of Health Studies, Western University, London, Canada; Health and Rehabilitation Sciences Program, Western University, London, Canada; Department of Family Medicine, Western University, London, Canada; School of Kinesiology, Western University, London, Canada; Section of Hospital Pediatrics & Pediatric Centre for Weight & Health, Alberta Children’s Hospital, Calgary, Canada; School of Kinesiology, Lakehead University, Thunder Bay, Canada

**Keywords:** Childhood obesity, Community intervention, Family health, Program evaluation, RE-AIM, Quality of life, Feasibility, Group dynamics

## Abstract

**Background:**

Increasing rates of childhood overweight and obesity highlight a need for the evaluation of lifestyle interventions. The purpose of the study was to determine the Reach, Effectiveness, Adoption, Implementation and Maintenance of a novel family-focused program targeting children with obesity (i.e., the Children’s Health and Activity Modification Program [C.H.A.M.P.]) using the RE-AIM framework, an evaluation tool for community-based health interventions.

**Methods:**

A single-centre, single cohort interventional feasibility study was conducted over the course of two years. Children with obesity and their families completed a 4-week group-based lifestyle intervention in Year 1 (*n* = 15; *M*_age_ = 10.6; 53% female) and/or Year 2 (*n* = 25; *M*_age_ = 10.6; 56% female). Outcome variables were measured pre- and post-intervention, as well as 6- and 12-months following completion of the formal program.

**Results:**

Overall, C.H.A.M.P. had high *reach* in terms of participant representativeness. In addition, participation in the program was associated with significantly improved standardized body mass index (BMI-*z*), body fat percentage, lean mass percentage, and child- and parent-proxy reported quality of life (QOL; *effectiveness*/*individual maintenance*). Furthermore, a number of community partnerships were built, strengthened, and maintained prior to, during, and following implementation of the two-year program (*adoption*/*setting maintenance,* respectively). Finally, the intervention was delivered as intended as evidenced by high adherence to the schedule, attendance rates, and cost effectiveness (*implementation*).

**Conclusions:**

Based on RE-AIM metrics, C.H.A.M.P. appears to be a promising childhood obesity program. The findings reported will inform researchers and practitioners on how to design and implement future community-based programs addressing pediatric obesity.

**Trial registration:**

ISRCTN Registry, Study ID ISRCTN13143236. Registered 27 March 2015.

## Background

Childhood obesity has reached epidemic proportions and is a major public health issue worldwide [1]. Among Canadian children and youth aged 5-17, it is estimated that the combined obesity and overweight prevalence is nearly 33% [2]. Not only is the growing prevalence a cause for concern, but the associated health implications of obesity place a significant burden on the health care system and can attenuate quality of life among those affected [3,4].

Multi-faceted treatment interventions integrating a family-based component have elicited significant and positive results among children with obesity [5-7]. Generally speaking, there appears to be consensus in the literature with regard to the components that should be included in comprehensive programs targeting childhood obesity. In particular, a focus on dietary change, the promotion of physical activity, behavioral counseling, and to parent training and modeling have been recommended [5,8-10].

In their systematic review of obesity treatment interventions, Oude Luttikhuis and colleagues [9] examined 64 childhood obesity intervention programs (54 randomized controlled trials [RCTs] and 10 drug RCTs). The authors found that combined dietary, physical activity, and behavioral therapy interventions were associated with reductions in level of overweight among children up to one year post-intervention, and involving families appeared to improve program effectiveness. These findings have been supported via recent reviews e.g., [11], supporting the conclusion that childhood obesity is a complex problem requiring a comprehensive treatment approach [9].

Interestingly, while multi-component e.g., [12] behavior-based lifestyle interventions targeting childhood obesity and integrating the family have demonstrated efficacy e.g., [13], many have taken place in school or clinical settings [14], have elicited variable outcomes, and have been costly with finite reach [6]. In addition, a number of community-based interventions have sought to evaluate program effectiveness using clinical indicators, behavioral outcomes, and psychosocial indices e.g., [6,7]; however, very few childhood obesity treatment studies have reported on other key elements that are required to facilitate the translation of results to community settings [14]. Such factors, related to study generalizability and dissemination, are addressed in the RE-AIM framework [15] which outlines measures for the Reach, Effectiveness/Efficacy, Adoption, Implementation and Maintenance of health promotion programs. RE-AIM provides a systematic and widely accepted means to determine the impact of programs on the basis of these five evaluative dimensions [15]; this is useful and arguably essential, as many pediatric obesity treatment studies have focused primarily on internal validity without considering external validity and the context of the interventions [14]. Furthermore, RE-AIM provides researchers with a guide for reporting intervention-related information that if reported consistently across studies, could have important implications for health professionals who are tasked with making decisions and recommendations on the basis of the childhood obesity treatment literature [14-16].

A systematic review of the behavioral treatment of childhood obesity literature was conducted in 2012 to evaluate the extent to which external validity dimensions were reported [14]. Of the 77 controlled studies included in the review, 100% lacked full reporting of the RE-AIM components. Some components were reported fairly consistently (e.g., participant attrition rate; target audience description; staff expertise and training) or in all studies (i.e., participant inclusion/exclusion criteria). The least reported components included implementation of the intervention content; time required to deliver the intervention; costs; program sustainability; and participation rates for delivery staff, individual participants, and settings. It was also noted that a limited number of studies reported the use of a quality of life measure [14]. The current study aims to address these concerns by reporting, as comprehensively as possible, on each of the RE-AIM dimensions.

The Children’s Health and Activity Modification Program (hereafter referred to as C.H.A.M.P.), a family-based lifestyle intervention for children with obesity, was designed on the basis of group dynamics theory and in response to the paucity of accessible community- and theory-based childhood obesity programs involving children and their parents e.g., [17] (see Martin and colleagues [18] for a detailed description of the study development and protocol). Further, given the substantial body of research which suggests that the camp experience itself (i.e., the natural environment, full-day attendance, increased group interaction and support, leadership opportunies, etc.) is associated with positive youth development e.g., [19], a day-camp approach was used. C.H.A.M.P. represented a feasibility project that was offered to a small number of children and caregivers over a 2-year period. Feasibility studies “enable researchers to assess whether or not the ideas or findings can be shaped to be relevant and sustainable” [20], p. 453. Hence, the primary purpose of the current study was to use RE-AIM to evaluate the reach, effectiveness, adoption, implementation and maintenance of C.H.A.M.P. in an effort to conduct a preliminary investigation of the program. It was hypothesized that a systematic evaluation of this 2-year project would provide important information related to the effectiveness of the intervention, as well as recruitment and logistical issues associated with the implementation of a research-based program for children with obesity. It was also hypothesized that C.H.A.M.P. would be associated with both short- and longer-term improvements in children’s body composition and health-related quality of life.

## Methods

The current study was a single-centre, single cohort interventional feasibility study held over the course of two years. As noted above, the RE-AIM framework was used to determine the feasibility of C.H.A.M.P., a multi-faceted community-based lifestyle intervention for children with obesity and their families. Various aspects of the program were used as indicators of each of the RE-AIM dimensions, as outlined in the Measures section below.

### Participants

Families were recruited through advertisements placed in local media, posters displayed in libraries, community centers, hospitals, and family medical clinics, and physician referrals. The recruitment phase was approximately 3 weeks (Year 1; July 2008) and 3 months (Year 2; May-July 2009) in duration. The discrepancy in recruitment length between the two years was a result of administrative delays. Children in the London, Ontario census metropolitan area (population = ~ 474,800) were eligible to participate in the program if they: (a) were between the ages of 8 and 14; and (b) had a BMI-*z* greater than or equal to the 95^th^ percentile for their age and sex. Children were assessed by a pediatrician and cleared for exercise participation. A total of 36 (different) children and families agreed to participate in the program in Year 1 (*n* = 16; mean age = 10.6; 50% female) and/or Year 2 (*n* = 25; mean age = 10.6; 56% female); 5 children/families were eligible and chose to participate in both years. One child from Year 1 was removed from the program during the second week due to behavioral and family issues, three children from Year 1 dropped out at the 6-month follow up, and five children (one from Year 1 and four from Year 2) dropped out at the 12-month follow up. Written informed consent and assent were obtained from parents and children, respectively, prior to program involvement.

### Procedure

The project was a 4-week intervention delivered to two cohorts during the month of August over two years. C.H.A.M.P. included physical activity, dietary, and behavior modification components for children, as well as educational sessions reflecting similar content for families. Children attended camp on weekdays from 9am-4pm for four consecutive weeks, and family members (i.e., parents and/or guardians only) attended weekly group-based educational sessions on four consecutive Saturdays from 10am-2pm.

A number of program-specific trained staff (6 in Year 1, 10 in Year 2) and volunteers (7 in Year 1, 9 in Year 2) were involved in the implementation of the intervention as program counselors and assistants, respectively. Five counselors and one volunteer were involved in both years. Potential staff were recruited via word of mouth and advertisements at Western University and a local YMCA. Candidates who were short-listed based on their credentials and experiences underwent initial screening and a two-phased interview process led by three members of the research team. All counselors, once hired, were required to complete a police background check, CPR/First Aid training, and a one-week counselor training program led by the Principal Investigator and Project Coordinator. This training included: (a) a detailed overview of the program, C.H.A.M.P. counselor manual, and research processes; (b) fine-tuning of the 4-week schedule for children and parents; (c) brainstorming sessions around various program-related topics; and (d) a one-day life coaching workshop that focused on effective and supportive communication. All counselors were certified school teachers, university students, and/or employees at the program delivery settings (i.e., Western University, Canadian Centre for Activity and Aging, or the YMCA). Volunteers were university (graduate and undergraduate) students.

Three researchers were responsible for the collection of data and supervision of the program for both years. Counselors ran the child-based portion of the intervention (i.e., supervised the children and led most activities) and a number of guest speakers (e.g., dietitian, life coach, exercise physiologist, psychotherapist, anti-bullying representatives) facilitated the family-based sessions in addition to leading some camp sessions (i.e., nutrition and life coaching sessions). Two-hour “booster sessions” were offered once every two months for one year following the formal intervention. These sessions were created to maintain contact among children and family members and to re-iterate, emphasize, and provide new information and resources pertaining to behavior modification strategies, physical activity, and healthy food choices.

Although C.H.A.M.P. was funded externally, each family also paid a fee of $200.00 (CAD) upon entry into the program. Caregivers of potentially eligible children were informed of this fee when they contacted the researchers about the program (i.e., this information was not included on the recruitment posters). The fee contributed toward the cost of school bus transportation to and from camp and a one-month family membership at the YMCA. Ethical approval for all study procedures was obtained from The University of Western Ontario Research Ethics Board for Health Sciences Research Involving Human Subjects.

### Measures

In addition to a demographic survey completed by caregivers at the beginning of the 4-week intervention, children and family members completed a number of research assessments [18]. For the purpose of the present study, only data pertinent to the RE-AIM dimensions are presented and discussed.

#### Reach

The *reach* of an intervention measures the participation rates and representativeness of individuals who participate in a program [21]. To determine representativeness, participant demographics were compared to census demographics in London, Ontario [22]. In addition, records of inquiries about the program were used to analyze the participation rate and most effective recruitment methods.

#### Effectiveness and individual-level maintenance

Given that C.H.A.M.P. was a research-based program that was delivered on a day-to-day basis by counselors and volunteers in a “real world” camp setting, effectiveness (rather than efficacy) data are reported as part of RE-AIM and within the context of the current feasibility study.

The *effectiveness* and individual-level *maintenance* elements of RE-AIM measure the short- and longer-term impact of an intervention, respectively [21]. In relation to C.H.A.M.P., these dimensions were assessed using: (a) standardized body mass index (BMI-*z*), body fat percentage, and muscle percentage (using a DXA scanner [GE Lunar]); and (b) the Pediatric Quality of Life Inventory 4.0 (PEDS-QL 4.0; [23]), a reliable and valid measure of health-related quality of life (QOL) for children ages 8 to 12 [24]. The questionnaire included a segment that was completed by the child and a proxy report that one parent/guardian completed based on his or her perceptions of the child’s QOL. Both the child and parent proxy questionnaires consisted of the following subscales: 1) physical (*n* = 8 items); 2) emotional (*n* = 5 items); 3) social (*n* = 5 items); 4) and school (*n* = 5 items) functioning. Individual-level maintenance was also assessed using participant attrition [14,15].

Measurements were collected at baseline, post-intervention, 6-month, and 12-month follow-up assessments. For the purpose of the current study (and given the timeframe of the intervention and follow-up assessments), program *effectiveness* referred to the evaluation of the abovementioned variables from pre- to post-intervention, whereas individual-level *maintenance* referred to the evaluation of these variables from pre-intervention to the 6- and 12-month follow-up periods. Effectiveness and maintenance data were analyzed using a series of one-way repeated measures ANOVAs. Effect size values (η^2^) were also calculated, and values of 0.01, 0.06, and 0.14 were considered small, medium, and large, respectively [25]. Post-hoc analyses were conducted to determine whether there were statistical differences in the variables of interest. Analysis of the PEDS-QL 4.0 [23] required that the physical subscale be analyzed separately from the remaining three subscales [26]. Because C.H.A.M.P. took place when children were not in school, the school subscale was removed from analyses. Due to the small number of participants, outliers were checked by visually scanning the data [27]. Missing data were replaced using a series mean [27]. The 12-month follow-up scores (Year 1) for the five children who participated in both years of the program were replaced with the series mean to avoid duplication between these values and Year 2 baseline values.

#### Adoption

Adoption includes an assessment of the delivery settings (i.e., intervention locations) and the participation rate of delivery agents (i.e., individuals who delivered the intervention components) involved in the implementation of a program [15,28]. Adoption of C.H.A.M.P. was analyzed by: (a) providing an overview of the delivery settings, the use of these settings over the 2-year period, and their potential for translating the research program into practice; and (b) summing the number of individuals and/or community organizations in London, Ontario and surrounding areas that were involved in the implementation of the program. As such, this information (including descriptions of the delivery settings which typically appear in the Methods section) is provided in the Results section.

#### Implementation

*Implementation* is measured by determining whether the intervention was delivered as intended [23]. The analysis of the implementation of C.H.A.M.P. was four-pronged. First, the original C.H.A.M.P. schedules (i.e., for both the child- and family-based sessions), in addition to revisions and notes made by research personnel, were analyzed to determine the percentage of the planned intervention that was actually implemented. Second, at the end of each week of C.H.A.M.P., the children completed fidelity checks (i.e., quizzes pertaining to the information provided during the week, completed independently and on-site) to evaluate the degree to which the material being disseminated was retained [29]. The parents/guardians also completed weekly fidelity checks (i.e., quizzes completed independently and on-site) related specifically to material presented during the family sessions. All fidelity (i.e., receipt of treatment [29]) checks were graded for accuracy to provide an estimate of the percentage of knowledge retained. Third, a comparison between the original budgeted costs of C.H.A.M.P. and the actual costs of running the program was calculated. Finally, attendance was assessed using records for the child and family portions of the program.

#### Setting-level maintenance

In the present study, setting-level maintenance was assessed by calculating the percentage of community organizations (who participated in the first year of the intervention) that were both asked and agreed to participate in the second year of the program.

## Results

### Reach

**Figure 1:**
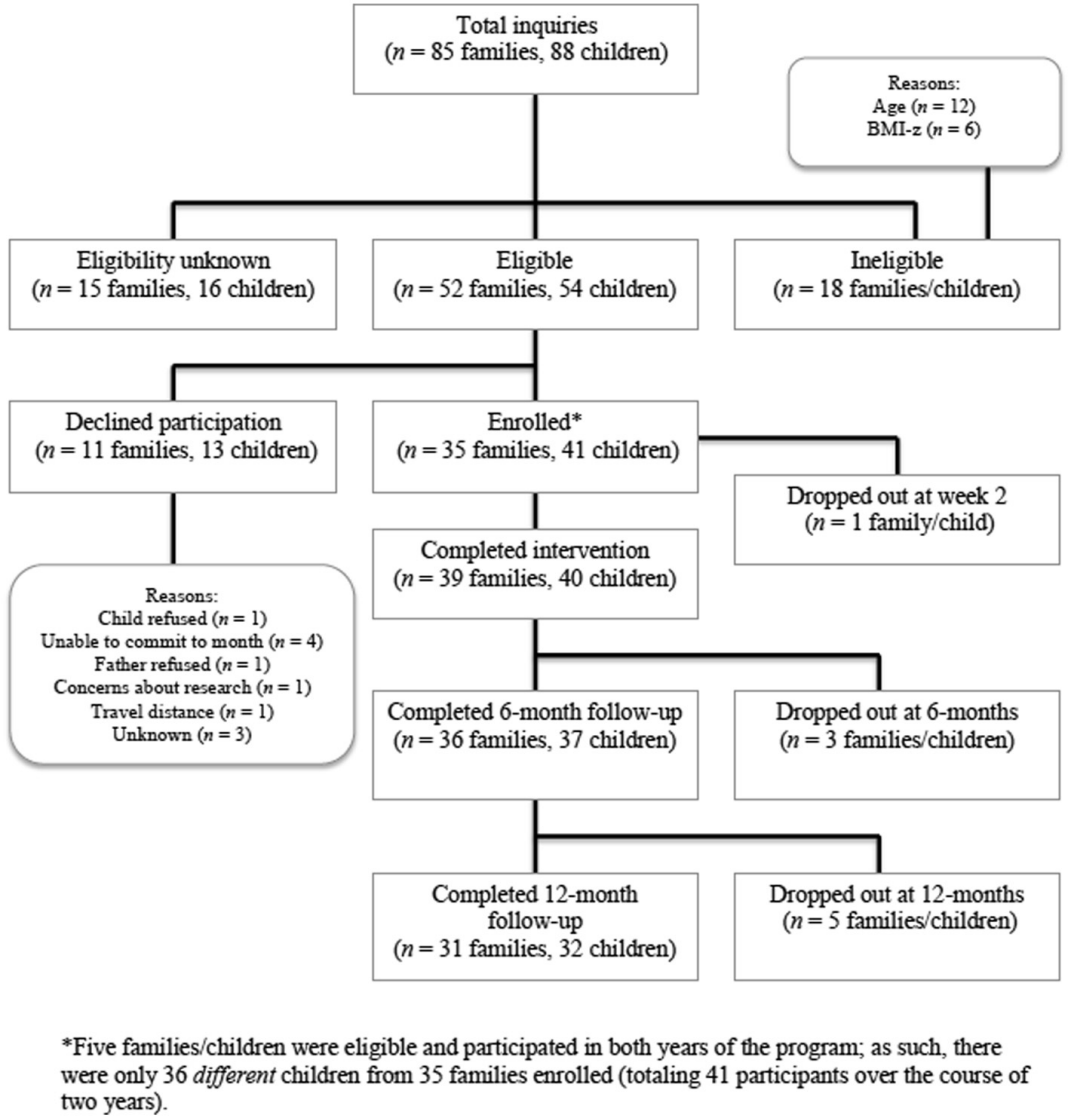
Combined (Years 1 and 2) participation and attrition rates of the Children’s Health and Activity Modification Program.

A total of 85 families contacted the researchers regarding the potential involvement of 88 children. See Figure 1 for an overview of the participation rates associated with the program (Years 1 and 2 combined). The reported number of children ages 5 to 14 living in London, Ontario in 2011 was 52,770 [22]. Unfortunately, data regarding the number of potentially eligible children in this age cohort were unavailable at the time of the study. Thus, bearing in mind that a substantial number of these children would not have been eligible for the program, C.H.A.M.P. reached approximately .07% of the individuals in this age cohort in the City of London (36 different children out of 52,770 in this age group). Seventy-four percent of children whose families contacted the research team and were deemed eligible received the intervention. Demographically, C.H.A.M.P. families were similar to the population from which they were drawn in terms of ethnicity (82.0% and 85.0% identified as Caucasian in C.H.A.M.P. and London, respectively), income (median income was $60-80,000 and $68,648 CAD, respectively), and employment status (93.2% and 93.0% employed, respectively).

In terms of total C.H.A.M.P. inquiries, the most successful means of recruitment were local newspaper advertisements (44.9%, *n* = 44 inquiries), posters (12.2%, *n* = 12), attendees from the previous year (7.14%, *n* = 7), word of mouth (6.0%, *n* = 6), radio advertisements (5.1%, *n* = 5), physician referrals (4.1%, *n* = 4), television interviews (2.0%, *n* = 2), and school board referrals (2.0%, *n* = 2). Sixteen (16.3%) families who inquired about the program did not disclose their source of program information. For families that participated in the program, the most successful means of recruitment were newspaper advertisements (52.2%, *n* = 23), word of mouth (18.2%, *n* = 8), posters (11.4%, *n* = 5), previous attendance (11.4%, *n* = 5), and radio advertisements (6.8%, *n* = 3). Several families heard about the program through more than one source.

### Effectiveness and individual-level maintenance

#### Body composition

Table 1 contains the descriptive statistics for body composition. BMI-*z* data showed a significant decrease over time along with a medium effect size (*F*(3,36) = 3.20, *p* < 0.05; η^2^ = 0.05). Post-hoc analyses revealed that BMI-*z* values decreased significantly from baseline to post-intervention and from baseline to the 6-month follow-up. Percentage of fat mass decreased significantly from baseline to post-intervention and was associated with a medium effect size (*F*(3,36) = 5.50, *p* < 0.05; η^2^ = 0.07), and percentage of muscle mass increased significantly from baseline to post-intervention with a large effect size (*F*(3,36) = 5.45, *p* < 0.05; η^2^ = 0.19). No changes in fat or muscle mass were sustained at the 6- or 12-month follow-up periods.

**Table 1:** **Descriptive and reliability statistics for health-related quality of life (QOL;** ***n*** **= 38)** ^**a**^ **and body composition (** ***n*** **= 39)** ^**b**^

**Measure**	**Pre-intervention**			**Post-intervention**			**6-month follow-up**			**12-month follow-up**		
	***M***	***SD***	***α***	***M***	***SD***	***α***	***M***	***SD***	***α***	***M***	***SD***	***α***
BMI-*z*	2.19	0.25	-	2.15*	0.25	-	2.14*	0.27	-	2.13	0.31	-
Lean mass (%)	53.84	4.11	-	54.82*	4.59	-	54.64	4.81	-	49.89	7.41	-
Fat mass (%)	45.24	4.06	-	44.22*	4.46	-	44.11	4.88	-	44.09	3.46	-
Child – physical QOL	73.39	14.16	0.94	79.61*	14.53	0.94	81.34*	14.58	0.94	79.44	14.97	0.94
Child – emotional QOL	66.05	19.94	0.94	75.66*	19.21	0.94	74.03	24.18	0.94	74.52*	19.84	0.94
Child – social QOL	65.00	21.34	0.94	70.79*	21.98	0.94	73.33*	21.34	0.94	71.94	18.81	0.94
Parent – physical QOL	66.81	16.03	0.94	82.04*	14.16	0.94	79.59*	17.02	0.94	80.40*	14.50	0.94
Parent – emotional QOL	60.92	19.72	0.94	76.45*	17.59	0.94	74.03*	24.18	0.94	71.54*	18.52	0.94
Parent – social QOL	58.16	22.13	0.94	79.86*	17.22	0.94	72.71*	23.43	0.94	76.65*	18.51	0.94

*Notes*. ^a^The Peds-QL 4.0 [23] was developed for children between the ages of 8-12. The maximum possible score on each of the subscales is 100. Two participants were removed from the QOL analyses as they were not in this age range.

^b^One child was removed from the body composition analyses because s/he was on a prescribed medication that is known to contribute to weight gain.

*Statistically significant change from baseline.

#### Quality of life

Table 1 also provides an overview of data pertaining to physical, emotional, and social QOL as measured by the PEDS-QL 4.0 [23].

#### Children’s reports

Children’s self-reported *physical* QOL increased significantly (from baseline to post-intervention, and from baseline to the 6-month follow up) and was associated with a large effect size (*F*(3,35) = 6.62, *p* < 0.05; η^2^ = 0.13). Children’s *emotional* QOL improved significantly from baseline to post-intervention and from baseline to 12-months and was associated with a medium to large effect size (*F*(3,35) = 4.38, *p* < 0.05; η^2^ = 0.11). Additionally, significant improvements were observed in children’s self-reported *social* QOL from baseline to post-intervention and from baseline to 6-months (*F*(3,35) = 4.64, *p* < 0.05; η^2^ = 0.11).

#### Parent proxy reports

Parents’ perceptions of their child’s *physical* QOL increased significantly from baseline to post-intervention, baseline to 6-months, and baseline to 12-months, and were associated with a large effect size (*F*(3,35) = 8.95, *p* < 0.05; η^2^ = 0.26). Parents’ proxy reported *emotional* QOL showed a significant increase from baseline to post-intervention, baseline to 6-months, and baseline to 12-months, and again, was associated with a large effect size (*F*(3,35) = 10.09, *p* < 0.05; η^2^ = 0.18). Similarly, parent’s perceptions of their child’s *social* QOL increased significantly from baseline to post-intervention, baseline to 6-months, and baseline to 12-months (*F*(3,35) = 14.55, *p* < 0.05) and were associated with a large effect (η^2^ = 0.32).

### Adoption

Four delivery settings were involved in the implementation of the program. In Year 1, the child-based portion of the program was held at the Canadian Centre for Activity and Aging, a not-for-profit research and education center at Western University, as well as a local YMCA. Anecdotal feedback from caregivers, children, and program staff suggested that the YMCA was fundamental in providing participants with a family-friendly location in which they could be physically active both during and outside of the intervention. Thus, it was selected as the “home base” for the second year of the program; the YMCA was also selected because of the potential for program sustainability at the community level. Additionally, a nearby field at a local high school was utilized for the outdoor components of the program in Year 2. All family-based sessions took place at Western University because of the availability of classrooms, audiovisual equipment, specialized learning resources (e.g., 3D anatomy lab), and free parking. All delivery settings were carefully selected, and in some cases, the delivery agents who assisted with the implementation of the program were recruited from these sites.

All of the delivery agents that were approached to participate in the child and/or family component(s) of the 4-week intervention agreed to do so (26 in Year 1, 23 in Year 2). The nature and extent of this participation varied and was divided into four categories: (1) guest presenters (42.9%; *n* = 12 individuals/organizations); (2) providing facilities to run the program (21.4%; *n* = 6 organizations); (3) field trips (25.0%; *n* = 7 organizations); and (4) providing resources for the program (10.7%; *n* = 3 organizations). Seven delivery agents participated in only one of the two years.

### Implementation

Overall, 92.8% of the originally planned activities for the children’s component of the intervention were implemented. New activities (i.e., those not planned for in the original schedule) were added on an as-needed basis. For the caregiver-focused portion of the program, 100.0% of the planned sessions were implemented.

Using the weekly “quizzes” as a measure of treatment fidelity, parents/guardians (*n* = 25) retained a mean of 66.8% of the information presented over the course of the 4-week intervention. The overall mean of information retained by the children over the 4 weeks was 72.0%.

The financial costs of C.H.A.M.P. were divided into three general categories: (1) salaries, wages, and benefits; (2) supplies and services; and (3) dissemination costs. The total *actual* cost of implementing the program (including all research-related expenses), for both years, was $141,642.32 (CAD). Salaries and benefits included salaries for the counselors, Project Coordinator, and health professionals involved in the delivery of the program (e.g., Psychotherapist and Registered Dietitian). Supplies and services included: project-related materials and supplies; transportation; advertisement costs; DXA scans and other research-related expenses; administration costs; capital purchases including a laptop, printer, computer software, and a license for the PEDS-QL 4.0 inventory; evaluation costs; and other costs including t-shirts, backpacks, binders, and prizes. Dissemination costs for C.H.A.M.P. included conference registration and travel. As noted above, the $200 fee paid by each family was spent on a one-month family membership at the YMCA that could be used outside of the program ($85 each); the remainder was used for bus transportation. See Table 2 for a detailed description of budgeted and actual costs for both years of the program.

**Table 2:** **C.H.A.M.P. budget: comparing budgeted versus actual expenditures for years 1 and 2 (combined)**

**Budget category**	**Original budget**	**Actual expenditures**	**Difference**	**Percentage of original budget**	**Percentage of actual expenditures**
Salaries and benefits	$96,732.00	$95,428.31	+$1,303.69	65.3%	67.3%
Supplies and services	$50,505.00	$45,189.99	+$5,315.01	34.1%	31.9%
Dissemination and travel	$1,000.00	$1,024.02	-$24.02	0.6%	0.7%
Total	$148,237.00	$141,642.32	+$6,594.68	–	–

*Note*. All costs listed are in Canadian dollars (CAD).

Insofar as attendance is concerned, the children’s overall attendance rate for the 4-week program was 91.0%. In total (for Years 1 and 2), the percentage of days attended was 93.3% in Week 1; 86.4% in Week 2; 93.8% in Week 3; and 90.3% in Week 4. In contrast, the overall mean attendance rate for parents was 69.2%. The percentage of weekend sessions attended by parents was 72.1% in Week 1; 53.6% in Week 2; 61.3% in Week 3; and 90.0% in Week 4.

### Setting-level maintenance

Twenty-three of the 26 original delivery agents involved in Year 1 of C.H.A.M.P. were approached for involvement in Year 2; 100% of these delivery agents agreed to participate. In addition, five new delivery agents agreed to participate in Year 2. New delivery agents were sought out as a means of improving the program based on feedback from children and families in Year 1.

## Discussion

The purpose of the study was to conduct a preliminary investigation of C.H.A.M.P. using a validated, systematic framework [15]. Using RE-AIM metrics, C.H.A.M.P. shows promise as a potentially effective and engaging childhood obesity program. These results are in line with previous findings related to participation in C.H.A.M.P.; namely, significant increases in physical activity self-efficacy among children [30], and positive perceptions of the program from both children [31] and parents [32]. The current study addresses an important gap in the literature on behavioral treatments of childhood obesity by addressing several key external validity factors [14]. Beyond these general observations, the following specific findings warrant further discussion.

C.H.A.M.P. addressed a number of barriers to healthy living by including supervised activities for children (including siblings) during caregiver sessions, providing transportation and free parking, and offering reduced pricing for a YMCA (family) membership following participation in the program. Moreover, many participants suggested that the camp format and culture were important in facilitating positive changes for children and families [32]. In addition, the low cost of C.H.A.M.P., relative to other summer camps for children, was well-received [32] and allowed families of all income levels to participate. It is important to note that the low cost of C.H.A.M.P. was possible because of extensive external funding; as such, the option of offering a comparable program at a similar price ($200.00 CAD for 4 weeks) may be unrealistic. Nevertheless, the abovementioned factors provide potentially useful context-specific information for the development of future pediatric obesity interventions. While a strength of our study was the detailed account of program costs [14], researchers should examine and analyze health care costs as well as other economic burden variables (e.g., caregiver time and productivity costs) related to childhood obesity in the future [33].

Results pertaining to the *effectiveness* and *individual-level maintenance* elements of RE-AIM showed that the children experienced significant improvements in fat and muscle mass from pre- to post-intervention, as well as a trend toward improvement at the 6- and 12-month follow-up assessments. In addition, BMI-*z* values decreased significantly from pre- to post-intervention, and these changes were sustained at 6-months following the intervention. In a similar vein, Owens and colleagues [34] used DXA scans to measure the body composition of children with obesity (aged 7 to 11), and found that fat and muscle mass improved after a 4-month physical activity intervention. However, no follow-up results after the formal intervention were reported. Interestingly, a meta-analysis of childhood obesity and overweight interventions conducted by Wilfley and colleagues [35] showed that children with obesity in control or enhanced-care control groups continued to gain weight during and after the intervention(s). In addition, a recent review performed by Franckle et al. [36] identified patterns of accelerated weight gain during the summer months (in comparison to the school year) for children and adolescents who were overweight or obese. Thus, the results of C.H.A.M.P., considered within the context of previous scientific findings, suggest that although some of the improvements in body composition were not sustained following the intervention, the program may have prevented further inevitable weight gain.

In their seminal paper on RE-AIM [15], Glasgow and colleagues noted the need for public health researchers to assess participant QOL outcomes in addition to physiological markers. In the current study, the short and long-term improvements in children’s physical, emotional, and social QOL are particularly noteworthy. Children’s self-reported emotional QOL increased following the intervention and was sustained at 12-months, while self-reported physical and social QOL increased and were sustained at 6-months following the intervention. Improvements in some QOL outcomes have also been documented in previous childhood obesity intervention research. For example, Robertson and colleagues [37] found that immediately following a 3-month family-based lifestyle intervention, children’s physical QOL improved, but their emotional and social QOL did not change. Of note is the fact that improvements in physical QOL among the children in the abovementioned study were not sustained at the 9-month follow-up [37]. With regard to caregiver perceptions of the child’s QOL, the parents in C.H.A.M.P. reported improvements in physical, emotional *and* social QOL following the intervention, and those improvements were all sustained at 12-months. Interestingly, while the parents in the Robertson et al. study reported improved perceptions of their children’s physical, emotional, and social QOL immediately following the intervention, only improvements in physical QOL were maintained. Despite these important findings, it would be worthwhile for future childhood obesity interventions to incorporate assessment periods that extend beyond 12 months [9,11].

Given that C.H.A.M.P. was a feasibility study and did not include a control arm, the effectiveness data presented above provide *preliminary evidence* that the intervention may improve children’s body composition and QOL. Despite the small number of participants, the moderate and large effect sizes reported for the significant findings offer consistent support for the effectiveness of the theory-based intervention, despite the small number of participants. However, more robust research designs, such as a pragmatic or randomized controlled trial [38], are needed to provide more definitive conclusions with regard to program effectiveness and efficacy. Also, in line with the primary focus of the study which was to evaluate several external validity-related factors in relation to C.H.A.M.P., eligible twin siblings were included in the program and analyses. In the future, additional consideration should be given regarding the inclusion of siblings in research- and community-based programs, and the potential randomization of only one family member to be included in analyses due to clustering effects.

With regard to *adoption*, C.H.A.M.P. was successful at building strong community partnerships which were vital to the success of the program. According to Estabrooks and colleagues [39], the inclusion of a variety of community-based organizations in the planning of a program aids in the development of lifestyle interventions that are attractive to both community members and health professionals who may deliver such programs. Additionally, support from local organizations increases the chances of building and sustaining successful programs [40].

Generally speaking, the *implementation* of C.H.A.M.P. was assessed by determining whether the program was delivered as intended. Although the percentage of planned activities implemented was high, treatment fidelity was moderate for both the children and parents. A variety of factors might have affected the proportion of knowledge retained including a possible lack of: (a) family commitment to the program; and/or (b) reinforcement of the lessons learned at C.H.A.M.P. while at home. Additionally, it is possible that too much information was provided to children and parents in a given week, or that the fidelity “quizzes” were overly comprehensive/difficult, poorly designed, or not ideally scheduled to facilitate recall/retention of information. In the future, interventionists should explore (and report) the use of additional methodological strategies (e.g., video monitoring, interviews with participants, etc.) to assess and improve the reliability and validity of behaviorally-based pediatric obesity interventions [29].

Although child attendance during the 4-week program was high, parental attendance during the family sessions was moderate. Despite this finding, our qualitative research has shown that many caregivers acknowledged the importance of role-modeling, and some expressed a desire for more involvement in the program (e.g., “…*There should be a C.H.A.M.P. camp for parents!*” [32], p. 119). The treatment of childhood obesity necessitates changing the lifestyle of the whole family [41]; thus, ensuring that families are ready to commit to an intervention is necessary. Given the growing body of evidence in support of caregiver-focused pediatric obesity interventions e.g., [42], next steps for our research team and others might be to: (1) examine and identify strategies and approaches that could be used to encourage and promote parental attendance and involvement; and (2) incorporate these strategies, along with the evidence-based group dynamics strategies that were successfully utilized in C.H.A.M.P. [18], in the design of a controlled trial targeting parents as the primary agents of change.

## Conclusions

Based on the thorough and systematic evaluation of C.H.A.M.P. using the RE-AIM framework, it can be concluded that this 4-week family-based program holds promise as a treatment intervention for children with obesity. Furthermore, the detailed reporting on several key elements within the RE-AIM dimensions provides important and pragmatic information that should assist researchers and practitioners in the design and implementation of future community-based pediatric obesity programs.
